# Primary fallopian tubal transitional cell carcinoma with exfoliation of malignant cells in cervical Pap smear

**DOI:** 10.1186/1742-6413-2-20

**Published:** 2005-12-09

**Authors:** Nalini Gupta, Radhika Srinivasan, Raje Nijhawan, Lakhbir Kaur Dhaliwal

**Affiliations:** 1 Department of Cytology and Gynecological Pathology, Post Graduate Institute of Medical Education and Research, Chandigarh, India; 2 Department of Cytology and Gynecological Pathology, Post Graduate Institute of Medical Education and Research, Chandigarh, India; 3 Department of Cytology and Gynecological Pathology, Post Graduate Institute of Medical Education and Research, Chandigarh, India; 4 Department of Obstetrics and Gynecology, Post Graduate Institute of Medical Education and Research, Chandigarh, India

Only 0.2 – 0.5% of primary female genital malignancies are tubal, and histologically most of these are adenocarcinomas. Primary transitional cell carcinoma (TCC) accounts for about 10% of primary tubal carcinomas. [1] Primary TCC of the fallopian tube with exfoliation of malignant cells in cervical Pap smear has not been described in the literature previously.

**Figure 1:**
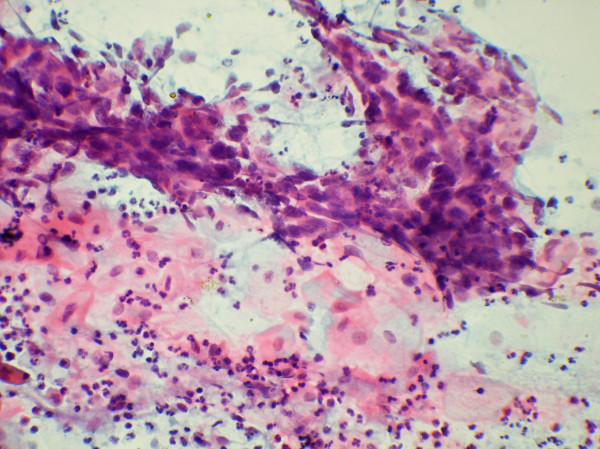
Clusters of malignant cells showing moderate pleomorphism in cervical Pap smear (Papanicolaou stain, #215; 250)

A 52-year- old lady presented with episodic spotting per vaginum. On general physical examination, the only significant finding was a 1.5 cm, firm, mobile, left supraclavicular lymph node. Fine needle aspiration (FNAC) from the left supraclavicular lymph node showed a metastatic carcinoma. The patient was investigated for detection of the primary malignancy. She was referred to the gynecologist, who took a cervical Pap smear. The Pap smear revealed mainly clusters as well as scattered cells showing moderate pleomorphism (Figure 1). The cells had moderate amount of cytoplasm, coarsely clumped granular chromatin and inconspicuous nucleoli. It was reported as positive for squamous cell carcinoma by the cytopathologist, who also advised colposcopy and biopsy confirmation. Colposcopical examination of the cervix was performed which showed no significant abnormality. A cervical biopsy and endocervical curettage were done. On microscopic examination, the endocervical curettage showed an occasional cluster of malignant cells entangled in mucus. The endocervical curettage was reported as suspicious of malignancy. The cervical biopsy was unremarkable. A repeat Pap smear also showed malignant cells as described previously. In view of the persistently positive Pap smear report, a total abdominal hysterectomy with bilateral salpingo-oophrectomy was carried out and the specimen was sent for histopathological examination.

**Figure 2:**
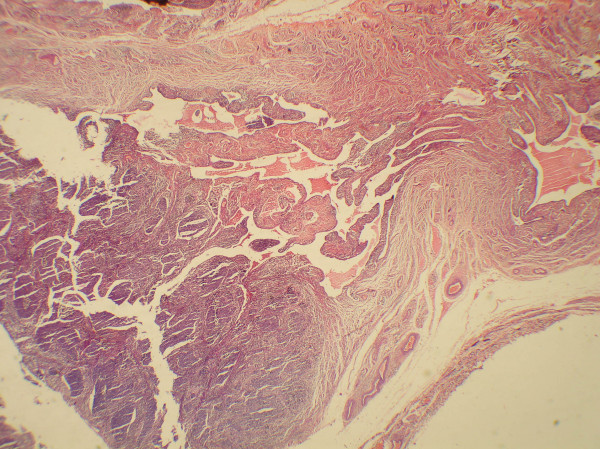
Microphotograph showing a tumor in the lumen of fallopian tube (H&E, 55).

Grossly, the cervix, endo- myometrium, bilateral ovaries and left fallopian tube were unremarkable. The right fallopian tube showed a nodule measuring about 2.5 × 2 × 2 cm occluding its fimbrial end. The cut section of this nodule was gray-white. Microscopically, a tumor was identified in the right fallopian tube. In one focus, a clump of tumor cells was seen in its lumen. The tumor showed large areas of necrosis. The tumor cells were arranged predominantly in ribbons, trabeculae and sheets (Figure 2). Histologicaly, this tumor pattern was that of a transitional cell carcinoma. The cells had "coffee bean" like nuclei and prominent nucleoli. There was no evidence of keratinisation. Mitosis was increased and the tumor was seen infiltrating transmurally. In addition, a small serosal tumor deposit was seen in the uterus.

Primary transitional cell carcinoma (TCC) of the fallopian tube is rare. The typical signs and symptoms of invasive tubal carcinoma include vaginal bleeding, clear or serosanguinous vaginal discharge, pelvic pain and a pelvic mass. The cervical Pap smear has shown exfoliated malignant cells rarely in cases of adenocarcinoma of fallopian tube. [1-5] Grossly, the tubal lumen is usually filled and dilated by papillary or solid and necrotic tumor. Tumor at the fimbriated end, with ready access to the peritoneal cavity, may also warrant individual staging. The morphology of TCC in fallopian tube is similar to TCC of urinary bladder. To the best of our knowledge, there are less than twenty cases reported of primary TCC in the fallopian tube in the English literature[6-8]. Primary TCC of fallopian tube showing exfoliated malignant cells in cervical Pap smear has not been described in the literature previously. Therefore, if the cervical Pap smear is positive for malignant cells and the cervical biopsy is negative, the patient should be investigated for a malignancy higher up in the gynecological tract. If endometrial curettage also does not reveal malignancy, the possibility of a tubal malignancy must be excluded by appropriate investigations.

**Figure 3:**
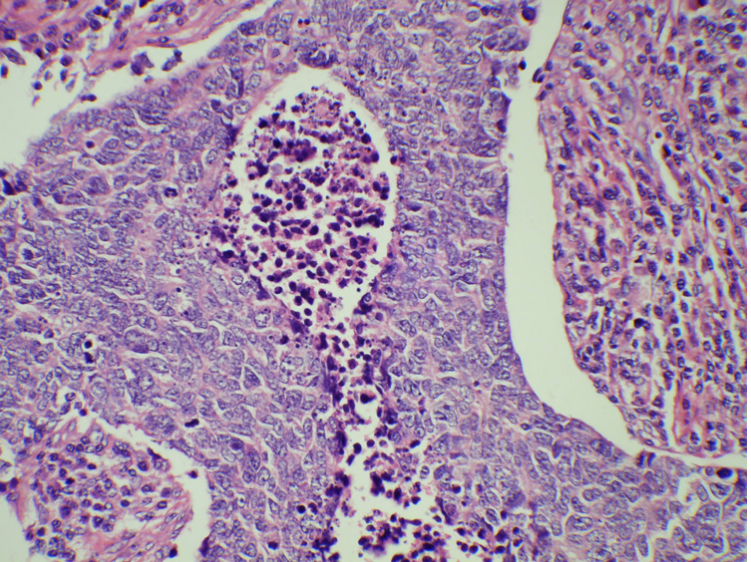
Microphotograph showing tumor cells arranged in ribbons and trabeculae with intervening areas of necrosis (Inset, H& E 250).
